# (2,2-Dichloro­vinyl)ferrocene

**DOI:** 10.1107/S1600536809006102

**Published:** 2009-02-28

**Authors:** Sébastien Clément, Laurent Guyard, Michael Knorr, Viktoria H. Gessner, Carsten Strohmann

**Affiliations:** a Institut UTINAM UMR CNRS 6213, Université de Franche-Comté, 16 Route de Gray, La Bouloie, 25030 Besançon, France; b Technische Universität Dortmund, Anorganische Chemie, Otto-Hahn-Strasse 6, D-44227 Dortmund, Germany

## Abstract

The title compound, [Fe(C_5_H_5_)(C_7_H_5_Cl_2_)], represents a versatile building block for the preparation of π-conjugated redox-active compounds or polymetallic organometallic systems due to the presence of the electrochemically active ferrocenyl unit. It is therefore a potential starting material for the preperation of the corresponding alkyne. In the crystal, the alkenyl unit and the cyclo­penta­dienide ring are almost parallel, with an angle between the best planes of only 10.6 (4)°.

## Related literature

The title compound was first prepared in 1963, see: Schloegl *et al.* (1963 ▶). For an alternative synthesis using a Corey–Fuchs route, see: Luo *et al.* (2000 ▶). For the preparation of some other halo-vinyl ferrocenes, see: Naskar *et al.* (2000 ▶). For related functionalized ferrocenes, see: Clément *et al.* (2007*a* ▶) and for [2.2]paracyclo­phanes, see: Clément *et al.* (2007*b* ▶). For the parent compound, ethenylferrocene, see: McAdam *et al.* (2008 ▶).
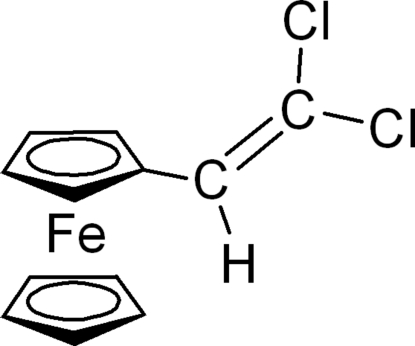


## Experimental

### 

#### Crystal data

[Fe(C_5_H_5_)(C_7_H_5_Cl_2_)]
*M* *_r_* = 280.95
Monoclinic, 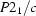

*a* = 14.340 (3) Å
*b* = 7.4370 (15) Å
*c* = 10.932 (2) Å
β = 108.48 (3)°
*V* = 1105.8 (4) Å^3^
*Z* = 4
Mo *K*α radiation
μ = 1.81 mm^−1^
*T* = 173 K
0.3 × 0.2 × 0.2 mm

#### Data collection

Bruker SMART APEX CCD diffractometer
Absorption correction: multi-scan (*SADABS*; Bruker, 1999 ▶) *T*_min_ = 0.594, *T*_max_ = 0.694
3293 measured reflections
1908 independent reflections
1372 reflections with *I* > 2σ(*I*)
*R*_int_ = 0.05

#### Refinement

*R*[*F* ^2^ > 2σ(*F* ^2^)] = 0.063
*wR*(*F* ^2^) = 0.174
*S* = 1.02
1908 reflections
136 parameters
H-atom parameters constrained
Δρ_max_ = 1.04 e Å^−3^
Δρ_min_ = −0.46 e Å^−3^

### 

Data collection: *SMART* (Bruker, 2001 ▶); cell refinement: *SAINT-Plus* (Bruker, 1999 ▶); data reduction: *SAINT-Plus*; program(s) used to solve structure: *SHELXS90* (Sheldrick, 2008 ▶); program(s) used to refine structure: *SHELXL97* (Sheldrick, 2008 ▶); molecular graphics: *ORTEP-3* (Farrugia, 1997 ▶); software used to prepare material for publication: *SHELXL97*.

## Supplementary Material

Crystal structure: contains datablocks I, global. DOI: 10.1107/S1600536809006102/zl2181sup1.cif

Structure factors: contains datablocks I. DOI: 10.1107/S1600536809006102/zl2181Isup2.hkl

Additional supplementary materials:  crystallographic information; 3D view; checkCIF report

## Figures and Tables

**Fig. 1.**
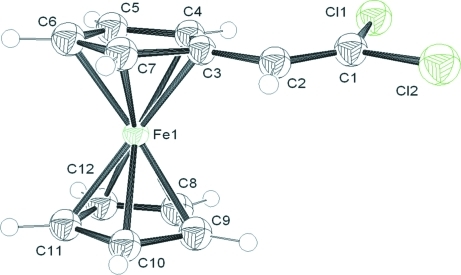
ORTEP presentation of (2) at the 30% probability level.

**Fig. 2.**
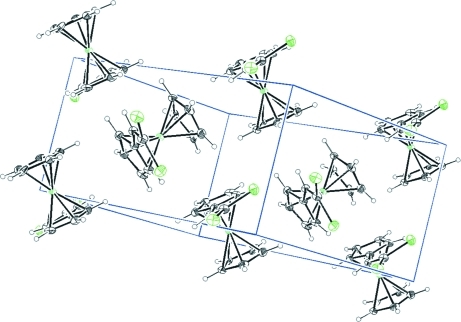
Packing diagramm of (2).

**Fig. 3.**
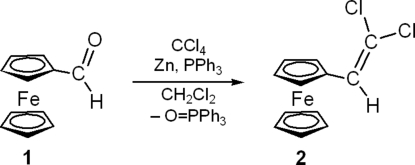
Synthesis of (2,2-Dichlorovinyl)ferrocene (2) under COREY-FUCHS reaction conditions.

## supplementary crystallographic information

### Comment

π-Conjugated ligands are widely applied in coordination chemistry, catalysis or polymer sciences. Among them, ethynylferrocene and its derivatives have gained special interest due to their use as building blocks for the synthesis of di- and polymetallic organometallic systems and advanced materials. In the context of our research into developing novel π-conjugated ligands such as functionalized ferrocenes (Clément *et al.*, 2007*a*) and [2.2]paracyclophanes (Clément *et al.*, 2007*b*) for potential applications in coordination chemistry, we have recently reported dehalobromation of (2,2-dibromovinyl)ferrocene and dibromovinyl[2.2]paracyclophane as a convenient route to the synthesis of the corresponding alkynes.

In order to take a closer glance on the influence of the halide leaving group in the starting materials {[Cl_2_C=C(H)—Fc] *versus* [Br_2_C=C(H)—Fc] (Fc = ferrocenyl)}, we prepared the title compound (2) according to a slightly modified literature procedure (Luo *et al.*, 2000) under Corey-Fuchs conditions by treatment of ferrocenecarbaldehyde (1) with CCl_4_ in the presence of zinc dust and triphenyl phosphane (Figure 3).

The molecular structure of 2 is shown in Figure 1. (2,2-Dichlorovinyl)ferrocene (2) crystallizes in the monoclinic crystal system, space group *P*2_1_/c. The two cyclopentadienyl rings are almost eclipsed with a mean cyclopentadienyl twist angle of 6.17°. The dihedral angle between the Cp ring planes is 0.1 (5)°. The bond distance of the vinylic double bond between C(1) and C(2) of 1.321 (9) Å is almost identical with that of (2,2-dibromovinyl)ferrocene [1.318 (4) Å]. The alkenyl unit and the cyclopentadienido ring are fairly coplanar with an angle between the two best planes [(C1 C2 Cl1 Cl2) and (C3 C4 C5 C6 C7)] of only 10.6 (4)°. This value determined for 2 is comparable to that determined for (2,2-dibromovinyl)ferrocene (10.43°) (Clément *et al.*, 2007*a*). Cl1 is involved in week C–H···Cl interactions (H8···Cl1^i^: 2.901 Å and C8–H8···Cl1^i^ 166.8°; symmetry operator i: x,-y+1/2,+z-1/2). Overall, it seems that the influence of the halide on the molecular geometry is negligeable. In contrast to the parent compound ethenylferrocene (McAdam *et al.*, 2008), where intermolecular C–H···π interactions are present in the solid state, no significant intermolecular interactions are observed in the packing of (2) (Figure 2).

### Experimental

(2,2-Dichlorovinyl)ferrocene (2): Triphenyl phosphane (2.40 g, 8.5 mmol), CCl_4_ (0.82 ml, 8.5 mmol) and zinc dust (0.55 g, 8.5 mmol) were placed in a Schlenk tube and 25 ml CH_2_Cl_2_ were slowly added. After stirring at room temperature for 28 h, ferrocenecarbaldehyde (1) (1.00 g, 4.24 mmol), dissolved in CH_2_Cl_2_ (10 ml), was added and stirring was continued for further 2 h. The reaction mixture was extracted with three 50 ml portions of pentane and CH_2_Cl_2_ was added when the reaction mixture became too viscous for further extractions. The extracts were filtered and evaporated under reduced pressure. The crude product was purified by column chromatography on silica gel with CH_2_Cl_2_/petroleum ether (1:1). Slow evaporation yielded red crystals of 2 (Yield: 91%). Characterization data have been previously described in the literature. (Luo *et al.*, 2000)

### Refinement

All H atoms were refined using a riding model in their ideal geometric positions. *U*_iso_(H) = -1.2U_eq_(C) was used for CH with C—H distances of 1.00 Å for the cyclopentadienyl H atoms and 0.95Å for the alkenyl hydrogen.

### Crystal data

**Table:**

[Fe(C_5_H_5_)(C_7_H_5_Cl_2_)]	*F*(000) = 568
*M**_r_* = 280.95	*D*_x_ = 1.688 Mg m^−^^3^
Monoclinic, *P*2_1_/*c*	Melting point: 382 K
Hall symbol: -P 2ybc	Mo *K*α radiation, λ = 0.71073 Å
*a* = 14.340 (3) Å	Cell parameters from 1338 reflections
*b* = 7.4370 (15) Å	θ = 1.5–25.0°
*c* = 10.932 (2) Å	µ = 1.81 mm^−^^1^
β = 108.48 (3)°	*T* = 173 K
*V* = 1105.8 (4) Å^3^	Irregular, red
*Z* = 4	0.3 × 0.2 × 0.2 mm

### Data collection

**Table:**

Bruker APEX CCD diffractometer	1908 independent reflections
Radiation source: fine-focus sealed tube	1372 reflections with *I* > 2σ(*I*)
graphite	*R*_int_ = 0.05
ω scans	θ_max_ = 25.0°, θ_min_ = 1.5°
Absorption correction: multi-scan (*SADABS*; Bruker, 1999)	*h* = −10→17
*T*_min_ = 0.594, *T*_max_ = 0.694	*k* = −8→7
3293 measured reflections	*l* = −12→11

### Refinement

**Table:**

Refinement on *F*^2^	Primary atom site location: structure-invariant direct methods
Least-squares matrix: full	Secondary atom site location: difference Fourier map
*R*[*F*^2^ > 2σ(*F*^2^)] = 0.063	Hydrogen site location: inferred from neighbouring sites
*wR*(*F*^2^) = 0.174	H-atom parameters constrained
*S* = 1.02	*w* = 1/[σ^2^(*F*_o_^2^) + (0.0967*P*)^2^ + 0.0275*P*] where *P* = (*F*_o_^2^ + 2*F*_c_^2^)/3
1908 reflections	(Δ/σ)_max_ < 0.001
136 parameters	Δρ_max_ = 1.04 e Å^−^^3^
0 restraints	Δρ_min_ = −0.46 e Å^−^^3^

### Special details

**Table:**

Geometry. All e.s.d.'s (except the e.s.d. in the dihedral angle between two l.s. planes) are estimated using the full covariance matrix. The cell e.s.d.'s are taken into account individually in the estimation of e.s.d.'s in distances, angles and torsion angles; correlations between e.s.d.'s in cell parameters are only used when they are defined by crystal symmetry. An approximate (isotropic) treatment of cell e.s.d.'s is used for estimating e.s.d.'s involving l.s. planes.
Refinement. Refinement of *F*^2^ against ALL reflections. The weighted *R*-factor *wR* and goodness of fit *S* are based on *F*^2^, conventional *R*-factors *R* are based on *F*, with *F* set to zero for negative *F*^2^. The threshold expression of *F*^2^ > σ(*F*^2^) is used only for calculating *R*-factors(gt) *etc*. and is not relevant to the choice of reflections for refinement. *R*-factors based on *F*^2^ are statistically about twice as large as those based on *F*, and *R*- factors based on ALL data will be even larger.

### Fractional atomic coordinates and isotropic or equivalent isotropic displacement parameters (Å^2^)

**Table:**

	*x*	*y*	*z*	*U*_iso_*/*U*_eq_	
C1	0.3941 (5)	0.1149 (9)	1.0606 (6)	0.0309 (16)	
C2	0.3017 (5)	0.0654 (9)	1.0059 (6)	0.0286 (16)	
H2	0.2812	−0.0326	1.0468	0.034*	
C3	0.2268 (5)	0.1356 (9)	0.8940 (6)	0.0262 (15)	
C4	0.2322 (5)	0.2584 (8)	0.7962 (6)	0.0297 (16)	
H4	0.2932	0.3182	0.7905	0.036*	
C5	0.1361 (6)	0.2818 (9)	0.7090 (7)	0.0339 (17)	
H5	0.1177	0.3602	0.6306	0.041*	
C6	0.0706 (5)	0.1740 (10)	0.7506 (7)	0.0350 (18)	
H6	−0.0018	0.1629	0.7067	0.042*	
C7	0.1254 (5)	0.0827 (10)	0.8631 (6)	0.0319 (17)	
H7	0.0985	−0.0039	0.9133	0.038*	
C8	0.2636 (6)	−0.0694 (10)	0.6107 (7)	0.0389 (19)	
H8	0.32	−0.0011	0.5981	0.047*	
C9	0.2703 (6)	−0.1880 (10)	0.7137 (8)	0.0372 (18)	
H9	0.3317	−0.2198	0.7847	0.045*	
C10	0.1753 (5)	−0.2558 (9)	0.6980 (7)	0.0320 (17)	
H10	0.1576	−0.343	0.7567	0.038*	
C11	0.1090 (5)	−0.1753 (9)	0.5874 (7)	0.0327 (17)	
H11	0.0365	−0.1969	0.5529	0.039*	
C12	0.1664 (5)	−0.0579 (11)	0.5309 (6)	0.0366 (19)	
H12	0.1408	0.0166	0.4511	0.044*	
Cl1	0.45229 (14)	0.2850 (3)	1.00576 (19)	0.0410 (5)	
Cl2	0.46791 (14)	0.0177 (3)	1.20093 (18)	0.0452 (6)	
Fe1	0.17755 (7)	0.01793 (12)	0.71562 (9)	0.0219 (3)	

### Atomic displacement parameters (Å^2^)

**Table:**

	*U*^11^	*U*^22^	*U*^33^	*U*^12^	*U*^13^	*U*^23^
C1	0.031 (4)	0.032 (4)	0.022 (4)	0.001 (3)	−0.002 (3)	−0.003 (3)
C2	0.030 (4)	0.029 (4)	0.026 (4)	−0.001 (3)	0.007 (3)	0.000 (3)
C3	0.030 (4)	0.023 (4)	0.021 (3)	0.001 (3)	0.002 (3)	−0.005 (3)
C4	0.034 (4)	0.024 (4)	0.023 (3)	−0.003 (3)	−0.003 (3)	−0.001 (3)
C5	0.039 (4)	0.024 (4)	0.029 (4)	0.007 (3)	−0.002 (3)	−0.006 (3)
C6	0.030 (4)	0.039 (5)	0.034 (4)	0.008 (3)	0.007 (3)	−0.005 (3)
C7	0.036 (4)	0.036 (4)	0.023 (4)	−0.003 (3)	0.009 (3)	−0.007 (3)
C8	0.043 (5)	0.037 (4)	0.041 (5)	−0.004 (4)	0.021 (4)	−0.011 (3)
C9	0.029 (4)	0.034 (4)	0.047 (5)	0.011 (3)	0.008 (4)	−0.002 (3)
C10	0.035 (4)	0.022 (4)	0.041 (4)	−0.002 (3)	0.015 (4)	−0.009 (3)
C11	0.025 (4)	0.036 (4)	0.035 (4)	−0.008 (3)	0.006 (3)	−0.016 (3)
C12	0.034 (4)	0.054 (5)	0.020 (4)	0.012 (4)	0.006 (3)	−0.011 (3)
Cl1	0.0320 (10)	0.0469 (12)	0.0410 (10)	−0.0091 (9)	0.0072 (8)	−0.0002 (9)
Cl2	0.0374 (12)	0.0488 (13)	0.0356 (10)	0.0041 (9)	−0.0081 (9)	0.0021 (8)
Fe1	0.0202 (5)	0.0199 (6)	0.0227 (5)	−0.0013 (4)	0.0026 (4)	−0.0025 (4)

### Geometric parameters (Å, °)

**Table:**

C1—C2	1.321 (9)	C7—Fe1	2.038 (7)
C1—Cl1	1.725 (7)	C7—H7	1
C1—Cl2	1.723 (7)	C8—C12	1.393 (10)
C2—C3	1.446 (9)	C8—C9	1.410 (10)
C2—H2	0.95	C8—Fe1	2.040 (7)
C3—C4	1.427 (9)	C8—H8	1
C3—C7	1.438 (10)	C9—C10	1.412 (10)
C3—Fe1	2.048 (6)	C9—Fe1	2.033 (7)
C4—C5	1.416 (10)	C9—H9	1
C4—Fe1	2.038 (6)	C10—C11	1.412 (10)
C4—H4	1	C10—Fe1	2.044 (6)
C5—C6	1.415 (10)	C10—H10	1
C5—Fe1	2.045 (7)	C11—C12	1.465 (10)
C5—H5	1	C11—Fe1	2.030 (7)
C6—C7	1.407 (9)	C11—H11	1
C6—Fe1	2.054 (7)	C12—Fe1	2.054 (6)
C6—H6	1	C12—H12	1
			
C2—C1—Cl1	125.0 (6)	C12—C11—Fe1	69.8 (4)
C2—C1—Cl2	122.1 (6)	C10—C11—H11	126.3
Cl1—C1—Cl2	112.9 (4)	C12—C11—H11	126.3
C1—C2—C3	130.7 (7)	Fe1—C11—H11	126.3
C1—C2—H2	114.6	C8—C12—C11	106.5 (7)
C3—C2—H2	114.6	C8—C12—Fe1	69.6 (4)
C4—C3—C2	131.5 (7)	C11—C12—Fe1	68.1 (4)
C4—C3—C7	106.8 (6)	C8—C12—H12	126.8
C2—C3—C7	121.7 (6)	C11—C12—H12	126.7
C4—C3—Fe1	69.2 (4)	Fe1—C12—H12	126.7
C2—C3—Fe1	126.2 (5)	C11—Fe1—C9	68.6 (3)
C7—C3—Fe1	69.0 (4)	C11—Fe1—C4	162.8 (3)
C5—C4—C3	108.1 (6)	C9—Fe1—C4	120.2 (3)
C5—C4—Fe1	70.0 (4)	C11—Fe1—C10	40.6 (3)
C3—C4—Fe1	69.9 (4)	C9—Fe1—C10	40.5 (3)
C5—C4—H4	125.9	C4—Fe1—C10	155.2 (3)
C3—C4—H4	126	C11—Fe1—C7	119.6 (3)
Fe1—C4—H4	126	C9—Fe1—C7	126.3 (3)
C6—C5—C4	108.5 (6)	C4—Fe1—C7	68.8 (3)
C6—C5—Fe1	70.1 (4)	C10—Fe1—C7	108.1 (3)
C4—C5—Fe1	69.4 (4)	C11—Fe1—C8	68.5 (3)
C6—C5—H5	125.7	C9—Fe1—C8	40.5 (3)
C4—C5—H5	125.7	C4—Fe1—C8	107.7 (3)
Fe1—C5—H5	125.7	C10—Fe1—C8	67.9 (3)
C5—C6—C7	108.1 (6)	C7—Fe1—C8	163.6 (3)
C5—C6—Fe1	69.5 (4)	C11—Fe1—C12	42.0 (3)
C7—C6—Fe1	69.3 (4)	C9—Fe1—C12	68.3 (3)
C5—C6—H6	126	C4—Fe1—C12	124.4 (3)
C7—C6—H6	126	C10—Fe1—C12	68.9 (3)
Fe1—C6—H6	126	C7—Fe1—C12	155.4 (3)
C6—C7—C3	108.5 (6)	C8—Fe1—C12	39.8 (3)
C6—C7—Fe1	70.5 (4)	C11—Fe1—C5	125.7 (3)
C3—C7—Fe1	69.8 (4)	C9—Fe1—C5	155.0 (3)
C6—C7—H7	125.8	C4—Fe1—C5	40.6 (3)
C3—C7—H7	125.8	C10—Fe1—C5	163.0 (3)
Fe1—C7—H7	125.8	C7—Fe1—C5	68.0 (3)
C12—C8—C9	109.8 (7)	C8—Fe1—C5	120.4 (3)
C12—C8—Fe1	70.6 (4)	C12—Fe1—C5	107.3 (3)
C9—C8—Fe1	69.5 (4)	C11—Fe1—C3	154.8 (3)
C12—C8—H8	125.1	C9—Fe1—C3	107.6 (3)
C9—C8—H8	125.1	C4—Fe1—C3	40.9 (3)
Fe1—C8—H8	125.1	C10—Fe1—C3	120.4 (3)
C8—C9—C10	108.0 (7)	C7—Fe1—C3	41.2 (3)
C8—C9—Fe1	70.0 (4)	C8—Fe1—C3	125.8 (3)
C10—C9—Fe1	70.2 (4)	C12—Fe1—C3	161.7 (3)
C8—C9—H9	126	C5—Fe1—C3	68.4 (3)
C10—C9—H9	126	C11—Fe1—C6	107.6 (3)
Fe1—C9—H9	126	C9—Fe1—C6	163.2 (3)
C9—C10—C11	108.3 (7)	C4—Fe1—C6	68.3 (3)
C9—C10—Fe1	69.3 (4)	C10—Fe1—C6	126.1 (3)
C11—C10—Fe1	69.2 (4)	C7—Fe1—C6	40.2 (3)
C9—C10—H10	125.8	C8—Fe1—C6	154.9 (3)
C11—C10—H10	125.8	C12—Fe1—C6	120.5 (3)
Fe1—C10—H10	125.8	C5—Fe1—C6	40.4 (3)
C10—C11—C12	107.4 (6)	C3—Fe1—C6	68.5 (3)
C10—C11—Fe1	70.2 (4)		
